# Post-translational activation of the C-terminus of polypeptides for the synthesis of peptide thioesters and peptide thioester surrogates

**DOI:** 10.3389/fchem.2024.1424953

**Published:** 2024-07-15

**Authors:** Yanbo Liu, Yasuhiro Kajihara, Ryo Okamoto

**Affiliations:** ^1^ Department of Chemistry, Graduate School of Science, Osaka University, Osaka, Japan; ^2^ Forefront Research Center, Osaka University, Osaka, Japan

**Keywords:** protein synthesis, semisynthesis, thioester, cysteine, chemical activation

## Abstract

Semisynthesis using recombinant polypeptides is a powerful approach for the synthesis of proteins having a variety of modifications. Peptide thioesters, of which the peptide C-terminus is activated by a thioester, are utilized for coupling peptide building blocks. Biological methods employing intein have been a center for the C-terminal thioesterification of recombinant polypeptides. Chemical activation has emerged as an alternative methodology for synthesizing peptide thioesters from recombinant polypeptides. Chemical reactions are compatible with various solutions containing organic solvents, chaotropic reagents, or detergents that are generally incompatible with biomolecules such as intein. Despite the potential utility of chemical activation, available methods remain limited. This article introduces the methods for the chemical activation of a peptide C-terminus applied to the chemical synthesis of proteins. By showcasing these methodologies, we aim to accelerate the advancement of new chemical reactions and methodologies and broaden the frontiers for the chemical synthesis of proteins.

## 1 Introduction

Semisynthesis using recombinant polypeptides is a powerful approach for the synthesis of proteins. Advances in biological techniques enable providing recombinant polypeptides as a building block for the chemical synthesis of proteins. By combining chemically synthesized peptide building blocks, semisynthesis has demonstrated the synthesis of various modified proteins such as histone proteins (Holt and Muir, 2015; Sun et al., 2022; Tashiro et al., 2022), glycoproteins (Reif et al., 2014; Reif et al., 2021; Ye et al., 2021; Zhao et al., 2022), and ubiquitinated proteins (McGinty et al., 2009; Fierz et al., 2012) with structurally defined forms.

C-terminal activation of recombinant polypeptides is one of the key steps to implement semisynthesis of proteins. As represented by peptide thioesters (Hojo and Aimoto, 1992; Dawson et al., 1994), C-terminally activated peptides play central roles in the coupling of peptide building blocks, affording full-length polypeptides of target proteins. The intein system has been a center for the preparation of peptide thioesters by recombinant technology (Muir et al., 1998; Muir, 2003). This system exploits the inherent function of inteins that induces protein splicing. Because this intrinsic activity on intein does not interfere with the biosynthetic pathway of proteins, a polypeptide with a built-in intein sequence can be produced as a peptide thioester form by recombinant techniques.

Chemical activation is an emerging approach for modifying the C-terminus of peptides. In the course of semisynthesis of proteins, a polypeptide is often designed as a building block. A large polypeptide segment (e.g., 50 amino acid residues<) of proteins often shows insoluble or aggregative characteristics as unfolded proteins. The aggregative characteristic of polypeptides potentially interferes with the correct folding of the intein moiety and the following thioesterification process. Chemical reactions are compatible with various solutions containing organic solvents, chaotropic reagents, or detergents. This feature significantly supports solubilizing polypeptide segments, and thereby, chemical activation is a potential approach for the preparation of peptide thioesters from recombinant polypeptides. However, despite the potential utility of chemical activation, available methods remain limited.

This minireview introduces the recent advances in the chemical activation of the peptide C-terminus, applied to the chemical synthesis of proteins. These methodologies are classified into i) N-S acyl shift-mediated activation and ii) Cys side-chain modification. By showcasing these methodologies, we aim to accelerate the advancement of new chemical reactions and methodologies and broaden the frontiers for the chemical synthesis of proteins.

## 2 N-S acyl shift-mediated activation

In recent decades, thioesterification methods mediated by N-S acyl shift activation have been well developed and utilized for Fmoc solid-phase peptide synthesis (Fmoc-SPPS). Because the thioester is unstable under the Fmoc-SPPS deprotection condition, the late-stage conversion of a thioester surrogate into a thioester derivative is usually needed. The N-S acyl shift, which initiates amide bond activation in protein splicing processes, also occurs under acidic conditions in the presence of the β-amino thiol motif. In 2005, both the Aimoto group (Kawakami et al., 2005) and the Melnyk group (Ollivier et al., 2005) reported peptide thioesterification methods under Fmoc-SPPS conditions based on an N-S acyl shift reaction. Following these pioneering attempts, various thioesterification methods using motifs such as N-alkyl cysteine (Hojo et al., 2007), N,N-bis(2-mercaptoethyl)amine (BSEA) (Ollivier et al., 2010), N-sulfanylethylanilide (SEAlide) (Sato et al., 2011), and cysteinyl-prolyl-ester (CPE) (Kawakami and Aimoto, 2007) were also developed.

**FIGURE 1:**
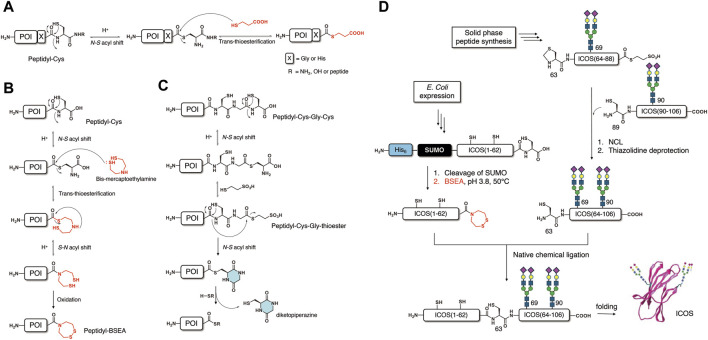
Thioesterification methods based on the N-S acyl shift. **(A)** Thioesterification via an N-S acyl shift at a Cys residue. **(B)** Activation of an X-Cys motif to form a BSEA amide derivative. **(C)** Activation of an X-Cys-Gly-Cys motif. **(D)** Semisynthesis of glycoprotein ICOS using a BSEA amide prepared using an N-S acyl shift.

In 2009, the Macmillan group developed a novel method of producing thioesters using a peptide derived from recombinant expression (Kang et al., 2009a). As a canonical amino acid containing the β-amino thiol motif, a cysteine is used to trigger an N-S acyl shift at the desired position. An Xaa-Cys-amide motif (Xaa = Gly or His, amide = NH_2_ or peptide) in a native peptide sequence could be activated to produce the corresponding Xaa-thioester via an N-S acyl shift under acidic conditions (Figure 1A).

Although this method was successfully applied to the production of both peptide thioester (Kang et al., 2009a; Kang et al., 2009b) and peptide hydrazide (Adams et al., 2013) as a thioester surrogate, the limited Xaa residue (Gly and His only) impeded the further application of this method. To prove the applicability of this method to the semisynthesis of proteins, the author synthesized an erythropoietin (EPO) 1–160 fragment’s hydrazide by recombinant expression and N-S acyl shift-mediated thioesterification (Adams et al., 2013). The obtained peptidyl hydrazide (1–160) was converted to thioester *in situ* for ligation with the C-terminal fragment (161–166) to yield the full-length non-glycosylated EPO. To broaden the substrate scope, the Macmillan group reported an optimized thioesterification method of activating the Xaa-Cys-OH motif at the C-terminal of the native peptide sequence (Cowper et al., 2015) (Figure 1A). Almost all amino acids they examined (Gly, Ser, Leu, Arg, and Phe) exhibited good reaction yield, except for valine. Notably, the main side reaction, the formation of aspartimide, could be mitigated after optimizing the reaction conditions. In 2014, the Macmillan group synthesized a cyclic peptide sunflower trypsin inhibitor (SFTI-1) by *E. coli* expression and the expressed peptide thioesterification method based on the N-S acyl shift (Shariff et al., 2014). A 14-residue peptide modified with C-terminal Cys was expressed as a fusion peptide with a His6 tag and thioredoxin. After cleavage of the fusion tag, activation of the C-terminal Cys was carried out via the N-S acyl shift to produce a thioester, which undergoes subsequent intramolecular cyclization to afford the desired SFTI-1 cyclic peptide. The development of these thioesterification methods via the N-S acyl shift blazed a new trail to obtaining modified or cyclic peptide/protein by recombinant expression in conjunction with chemical activation.

In 2021, the Okamoto and Kajihara group reported expressed peptide thioesterification methods using two motifs: 1) Xaa-Cys-OH and 2) Xaa-Cys-Gly-Cys-OH (Okamoto et al., 2021). In the first method (Figure 1B), they activated the Xaa-Cys-OH junction by an N-S acyl shift, followed by trans-thioesterification with bis-mercaptoethylamine (BSEA) producing peptidyl-BSEA. The subsequent formation of disulfide bonds under air oxidation could suppress potential reverse reactions and result in good reaction yield. The obtained peptidyl-BSEA could be utilized as a thioester surrogate, which is activated by the reduction of the disulfide bond to trigger an N-S acyl shift, as established by the Melnyk group. In the second method, which utilizes an Xaa-Cys-Gly-Cys-OH motif (Figure 1C), the thioesterification is carried out in two steps in a similar manner to the CPE method (Kawakami and Aimoto, 2007): 1) activation of the Gly-Cys junction by the N-S acyl shift to produce a Xaa-CG -thioester; 2) an N-S acyl shift at the Xaa-Cys junction followed by the formation of diketopiperazine (DKP) to produce a Xaa-DKP thioester. By using the Xaa-Cys-OH, the Okamoto and Kajihara group succeeded in the semisynthesis of homogeneous glycoprotein inducible T cell costimulatory (ICOS) (Okamoto et al., 2021). In this semisynthesis (Figure 1D), a peptidyl-BSEA containing 62 residues was efficiently prepared by recombinant expression and activation of Xaa-Cys-OH via an S-N acyl shift, as described above. The two C-terminal segments (63–88; 89–106) containing homogeneous diasialo-N-glycan (N69, N90) were synthesized by chemical synthesis based on SPPS. Sequential ligations using the expressed peptidyl-BSEA could afford full-length ICOS glycosyl-polypeptide, which was subjected to *in vitro* folding to afford folded ICOS with a native structure. By utilizing the expressed peptide thioesterification method, a protein with homogeneous post-translational modification could be obtained in a few chemical conversion steps.

Analogous to the N-S acyl shift reaction, an N-O acyl shift reaction could also be used to produce peptide thioester from recombinant expressions. In 2015, the Otaka group reported nickel (II)-mediated N-O acyl shift reactions at the naturally occurring sequence containing Ser/Thr-X-His-Z motif (X and Z ≠ proline) (Tsuda et al., 2015). Two sequential reactions, the formation of a square-planer nickel (II)-bound active complex and the N-O acyl shift, are incorporated in this method. The resultant peptidyl Ser/Thr-isoesters could be converted into peptidyl hydrazides as a thioester surrogate. Direct preparation of protein oxoesters has been demonstrated by using genetic code expansion (Li et al., 2012). These pre-modified recombinant proteins can also give peptide hydrazides through treatment with hydrazine.

## 3 Methods based on Cys side-chain modification

**FIGURE 2:**
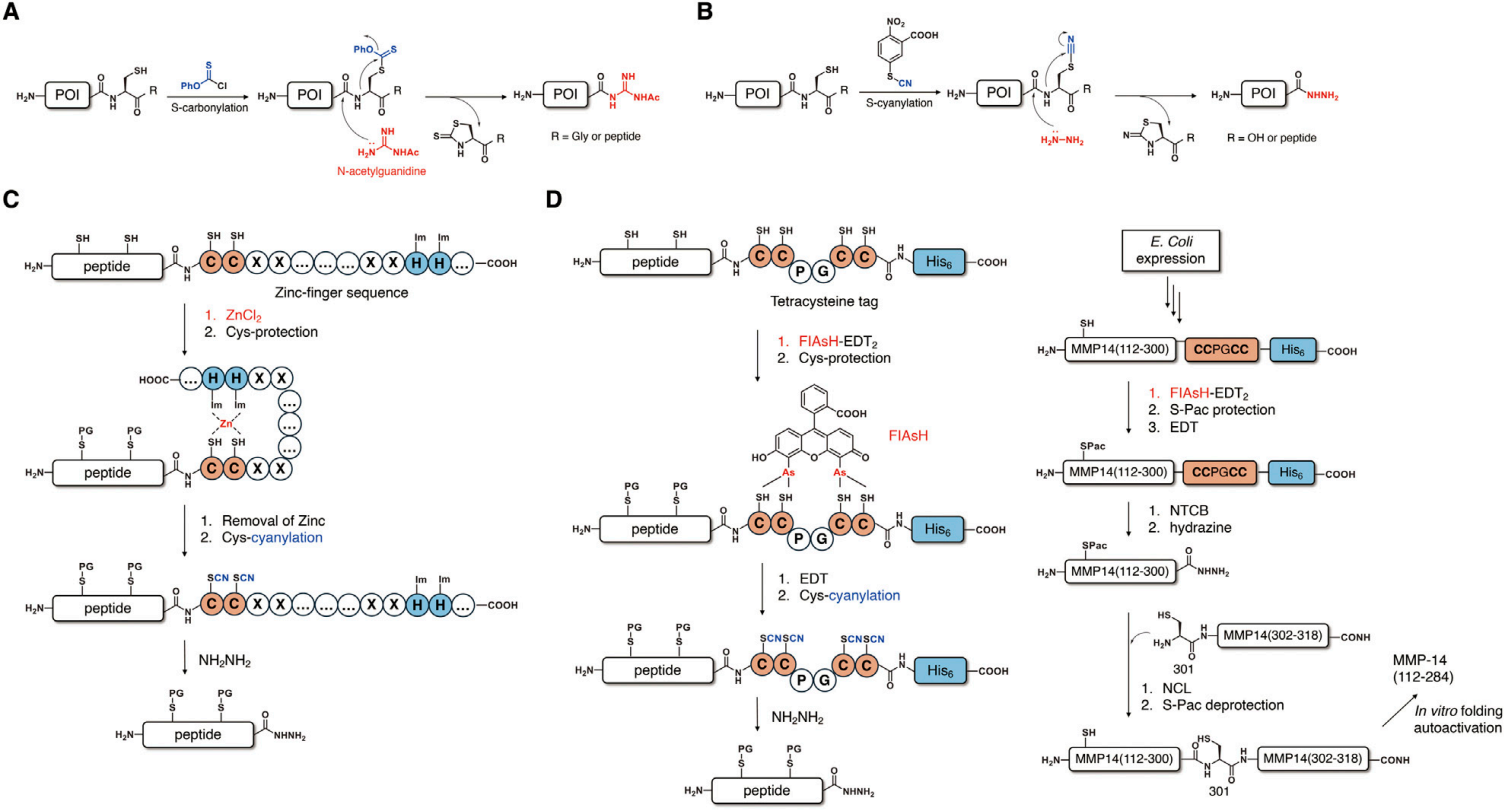
Thioesterification methods based on a Cys side-chain modification. **(A)** Cys-carbonylation and conversion to peptidyl-N-acetylguanidine and **(B)** Cys-cyanylation and conversion to peptide hydrazide. **(C)** Selective Cys-cyanylation via a zinc-finger sequence. **(D)** Selective Cys-cyanylation via coordination of the tetracysteine sequence and the FIAsH group.

Another approach to activating the amide bond at the Xaa-Cys junction was achieved by the modification of the Cys side chain. It was reported that the cyanylation of the cysteine side chain’s thiol could lead to an attack of cysteine’s amidic nitrogen on the cyano group, resulting in hydrolysis or aminolysis of the amide bond under the basic conditions (Degani and Patchornik, 2002). Based on this method, in 2012, Okamoto and coworkers reported a novel method of thioesterification of an unprotected peptide sequence by the S-carbonylation of cysteine’s thiol (Figures 2A) (Okamoto et al., 2012). The selective S-carbonylation of free cysteine residues enables activation of the Xaa-Cys-NHR (R = amino acid or peptide) motif by the nucleophilic attack of N-acetylguanidine by simultaneous N-carbonylation to produce the corresponding peptidyl N-acetylguanidine without significant epimerization. This method demonstrated the practical scope of a substrate employing an Xaa residue except for Lys, which requires Boc protection of its side chain. The resultant N-acetylguanidine derivatives could be further converted into thioesters for chemical ligation. It is worth noting that the peptidyl N-acetyl guanidine shows potential for kinetically controlled ligation (KCL) due to its stability against 4-mercaptophenylacetic acid (MPAA). Based on this method, the Okamoto and Kajihara group succeeded in the semisynthesis of glycoprotein interleukin-13 (IL-13) (Okamoto et al., 2014).

Extending the idea of activation of a peptide bond by Cys side-chain modification, the Kajihara group also developed another expressed peptide thioesterification method based on S-cyanylation (Figures 2B) (Kajihara et al., 2014; Chong et al., 2022). In this approach, the Xaa-Cys junction is activated by selective cyanylation of cysteine’s side chain using either 1-cyano-4-dimethylaminopyridium tetrafluoroborate (CDAP) or 2-nitro-5-thiocyanatobenzoic acid (NTCB), followed by the hydrazinolysis reaction under basic conditions to afford peptide hydrazide as a thioester surrogate. Peptide hydrazide, which is widely used in protein chemistry as a versatile precursor, could be activated through the formation of either peptidyl azide (Fang et al., 2011) or peptidyl pyrazole derivatives (Flood et al., 2018). Based on the S-cyanylation method, the Kajihara group prepared a polypeptide thioester (1–94) of interferon-γ by *E. coli* expression. Polypeptide (1-94)-Cys-His6 was obtained from recombinant expression and subjected to the expressed peptide thioesterification, which was carried out via Cys-cyanylation and hydrazinolysis. Activation of the resultant peptidyl hydrazide afforded peptidyl thioester, which could be used for the semisynthesis of full-length N97-glycosylated interferon-γ. The Kajihara group also achieved semisynthesis of full-length glycosyl-polypeptide interferon-β by using the same thioesterification method via Cys-cyanylation. In 2017, the Li group reported the semisynthesis of a murine prion protein by using this thioesterification method to obtain expressed peptide thioester (Shi et al., 2017). Recently, Liu and coworkers succeeded in the semisynthesis of ubiquitin protein conjugate by utilizing the S-cyanylation method (Qiao et al., 2020). In this research, they prepared ubiquitin (G76C)-His6, which contains a mutated cysteine residue for thioesterification via Cys-cyanylation by recombinant expression. The native ubiquitin sequence had no cysteine, and it had a G75 residue that exhibited a high reaction yield due to the lowest steric hindrance on the side chain. These examples make Cys-cyanylation a robust method for conjugating ubiquitin. Through the expressed peptide thioesterification, the obtained ubiquitin hydrazide could undergo the peptide–hydrazide ligation, which enables facile access to desired ubiquitin–protein conjugates.

Although the above-mentioned thioesterification approaches based on the modification of free cysteine have advantages in both milder conditions and broader substrate scope, their application was limited due to a lack of selectivity among cysteine residues. Several methods use additional external sequences to selectively modify and activate the Xaa-Cys motif at the desired position in the presence of other cysteines.

In 2016, the Otaka group reported selective S-cyanylation for the expressed peptide thioesterification by utilizing a zinc-finger sequence (Figure 2C) (Miyajima et al., 2016). In order to selectively activate the Xaa-Cys junction at the C-terminal of an unprotected peptide containing internal cysteines, a C2H2 type zinc-finger sequence containing a Cys-Asp-Ile-Cys (CDIC) motif is attached to the C-terminal of protein of interest (POI) during *E. coli* expression. The coordination of the zinc cation with the CDIC motif and the two histidines in the zinc-finger sequence serve as a temporary protection of its two cysteine residues and enables selective protection of native cysteine residues in the target peptide sequence by either a sulfonate group or a 6-nitroveratryl group. The coordinated zinc cation could be smoothly removed under acidic conditions via the eluent containing TFA during RP-HPLC processes, which exposes a free CDIC motif containing free cysteines for S-cyanylation. Finally, hydrazinolysis at the S-cyanylated cysteine, which is adjacent to the C-terminal of the peptide, could produce the desired peptide hydrazide. By using this method, Otaka and coworkers achieved regioselective cysteine modification and C-terminal activation in the presence of native internal cysteine residues.

In 2022, the He group reported another regioselective S-cyanylation method by attaching a tetracysteine sequence (CCPGCC-His6) to the C-terminal of the POI (Figure 2D) (Mo et al., 2022). In this method, they developed a 4′,5′-bis(1,3,2-dithioarsolan-2-yl)-fluorescein (FIAsH) group, which was inspired by a fluorescent labeling method pioneered by the Tsien group for selective coordination with the sequence (Griffin et al., 1998). The FlAsH group can temporarily protect C-terminal Cys residues and thereby facilitates selective protection of cysteine residues in the POI by the phenacyl (Pac) group. Subsequent deprotection of FIAsH by 1,2-ethanedithiol (EDT) could release a free cysteine adjacent to the C-terminal, which enables S-cyanylation and hydrazinolysis in a similar manner as the former method to afford peptidyl hydrazides. This novel method based on the coordination of the tetracysteine sequence and the FIAsH group has several advantages, including selective and reversible binding, as well as small tag size, which does not interfere with the recombinant expression of the POI. With this method in hand, He and coworkers succeeded in the semisynthesis of the catalytic (112–284) and hinge (285–318) domains of matrix metalloprotease 14 (MMP-14) (Figure 2C). In this semisynthesis, they obtained an expressed long peptide thioester (112–300) containing a native cysteine by selective C-terminal activation using the tetracysteine tag, including a one-pot three-step reaction (FIAsH coordination, Pac protection, and EDT treatment) and a one-pot two-step reaction (S-cyanylation and hydrazinolysis). The overall yield was approximately 25%, suggesting that this method was efficient toward difficult synthetic targets with a long peptide sequence. The obtained peptidyl hydrazide was used for NCL with a C-terminal fragment (302–318) to give a full-length MMP-14 polypeptide, which exhibited considerable catalytic activity after folding.

## 4 Enzymatic methods

In addition to the above-mentioned chemical methods for expressed peptide thioesterification/ligation, several enzymatic methods have also been developed and applied to the semisynthesis of proteins. In 2004, the Pollock group reported sortase-mediated protein ligation, which selectively ligates peptides with an LPXTG motif (Mao et al., 2004). The Pentelute group described protein thioester synthesis enabled by sortase in 2012 (Ling et al., 2012). In this research, they used sortase to recognize the LPXTG motif modified at the C-terminal of a POI and then converted it into an LPXTG-peptide thioester via a reaction with a peptide thioester having an N-terminal glycine. The Liu group demonstrated a sortase-mediated hydrazinolysis to produce LPXT-hydrazide at the C-terminal of the POI (Li et al., 2014). Notably, this reaction is irreversible because the product is no longer substrate recognized by sortase. Then, in 2015, the Liu group reported another enzyme that mediated enzymatic ligation at a specific sequence: butelase (Cao et al., 2015). Butelase recognizes the NHV motif and cleaves the amide bond between Asn and His, which enables the installation of glycinyl thioester to the C-terminal. In 2019, the Liu group reported the chemical ubiquitination of recombinant histone, which was accomplished by using Ub C-terminal hydrolase YUH1 to produce Ub-NHNH_2_ (Chu et al., 2019).

In 2019, the Otaka group reported a unique enzyme-mediated hydrazinolysis reaction to prepare expressed peptide thioester in a traceless manner (Komiya et al., 2019). The enzyme they used, namely, carboxypeptidase Y (CPaseY), exhibits the activity to hydrolyze one peptide bond through an O-acyl intermediate. The treatment with hydrazine in the presence of CPaseY could produce a corresponding peptidyl hydrazide as a thioester surrogate. However, the enzymatic activity of CPaseY also led to an over-reaction byproduct. To solve this problem, the author designed a Cys-Pro-Leu motif that modified the C-terminal of POI for the CPaseY-mediated thioesterification. Due to the recognition preference of CPaseY (Leu >> Pro), Cys-Pro-hydrazide is generated through a hydrazinolysis reaction without byproducts. Subsequent thioesterification and diketopiperazine formation led to traceless formation of a peptidyl thioester at the C-terminal of the POI. They further demonstrated that the CPaseY-mediated method could be utilized for hydrazinolysis reactions of repeating Cys-Pro motifs, which led to more efficient thioesterification regardless of the C-terminal residue of the POI (Ueda et al., 2020).

## 5 Conclusion and outlook

The development of the chemical C-terminal activation methods greatly facilitated thioesterification of expressed peptides, which enabled semisynthesis of target proteins in a few conversion steps. Notably, semisynthesis based on chemical C-terminal activation provides facile access to proteins with post-translational modification. Currently, chemical C-terminal activation methods are mainly based on the activation of the cysteine residue by either the N-S acyl shift or Cys side-chain modification. The selective activation of cysteine at the desired position in the presence of internal cysteine in a POI, which was achieved by attaching an external sequence such as the zin-finger sequence, might blaze a new path to the semisynthesis of large protein targets. Despite these achievements, the application of these chemical methods to the semisynthesis of proteins with post-translational modifications was still limited. We expect that the development of novel chemical C-terminal activation methods can inspire more attempts to semisynthesize homogeneous modified proteins, which is essential to the elucidation of their function.
